# Supplementary material for: Wind Power Persistence Characterized by Superstatistics
Source: Sci Rep. 2019 Dec 27;9:19971. doi: 10.1038/s41598-019-56286-1 (PMC6934744; doi:10.1038/s41598-019-56286-1)
Supplement: Supplementary file 1 — Supplementary Information [file 41598_2019_56286_MOESM1_ESM.pdf]

Supplementary Information

accompanying the manuscript

Wind Power Persistence Characterized by Superstatistics

by

Juliane Weber, Mark Reyers, Christian Beck, Marc Timme, Joaquim G. Pinto, Dirk Witthaut, and Benjamin Schäfer

This Supplementary Information discusses methods used in the main text in more detail and provides additional evidence for findings discussed therein. In particular, we provide a map displaying the European locations of which we analyze the wind data and present complementary (high- vs low-wind) statistics. Furthermore, we continue the superstatistical analysis, emphasizing that conditioning the data on f-parameters indeed reveals new insights. Also, we display individual snapshots of the synoptic analysis. In addition, we show that a different time resolution or the introduction of a cut-off wind velocity do not alter our results significantly. We highlight long-range correlations of the wind time series and extract the Hurst exponent. Finally, we estimate the increasing need for back-up storage when including heavy tails in the dimensioning considerations

SUPPLEMENTARY NOTE 1

European locations

The downscaled ERA-Interim data gives wind velocity statistics for the European region with a 0.11°resolution. To evaluate the data, we used both the full grid data over Europe, investigating for example the kurtosis of wind persistence statistics, but also analyzed individual locations. In particular, we chose 9 different locations to illustrate that q-exponentials are a better description to the data than exponentials. The locations of the measurement points are given in Supplementary Fig. 1, with special emphasis on Alpha Ventus and Harhaeuser Wald because we used these two locations to showcase the superstatistical approach. Furthermore, we list the longitude and latitude of those locations in Supplementary Table I.

Supplementary Table I. Geographical positions of locations used within the downscaled ERA-Interim data set. See Supplementary Fig. 1 for a European map showing these locations.

Location name	Latitude	Longitude
Alpha Ventus	54.01	6.38
EnbW Baltic2	54.59	13.84
Kemin Ajos	65.43	24.42
Harthaeuser Wald	49.15	9.25
Spain	42.98	-8.06
Southern France	43.47	2.54
Italy	40.98	15.42
Austria	48.59	16.18
Greece	37.96	21.95

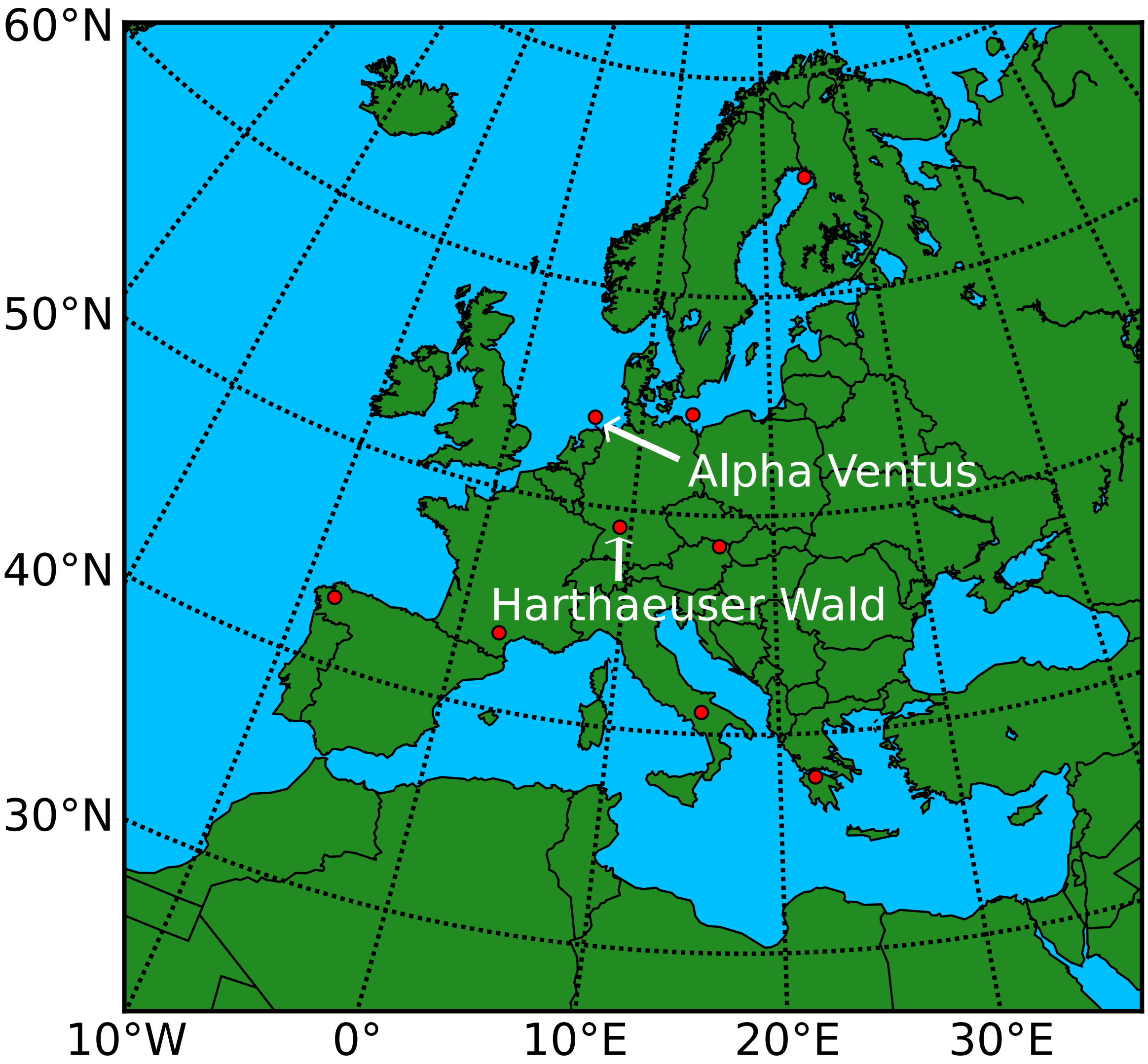

Supplementary Figure 1. **Positions of the selected locations for wind persistence analysis.** Red dots indicate selected locations that were chosen for visualizations of the ERA-Interim persistence analysis. Of special interest are Alpha Ventus, a German offshore wind farm in the North Sea, and data recorded at the wind farm Harthaeuser Wald (southern Germany) as they are representative for locations with high-wind speeds (Alpha Ventus) and locations of low-wind speeds (Harhaeuser Wald). The map was created using Python 2.7.12: `https://www.python.org/`.

SUPPLEMENTARY NOTE 2

Complementary high and low-wind analysis

We complement the analysis presented in the main text by investigating high-wind statistics for a low-wind location (here: Harthaeuser Wald) and low-wind statistics for a high-wind location (here: Alpha Ventus). Supplementary Fig. 2 gives the statistics for Harthaeuser Wald and Alpha Ventus for their atypical wind conditions, i.e., high-wind speeds at Harthaeuser Wald and low-wind speed at Alpha Ventus. Since the total number of these events is comparatively small, we refrain from splitting these into histograms, based on different CWT directions or f-parameters.

We recall the kurtosis as a function of the q-parameter from the main text as

$$\kappa_{q\text{-exp}} = \frac{9}{5} + \frac{81}{30-25q} + \frac{1}{q-2} + \frac{8}{4q-5}. \tag{1}$$

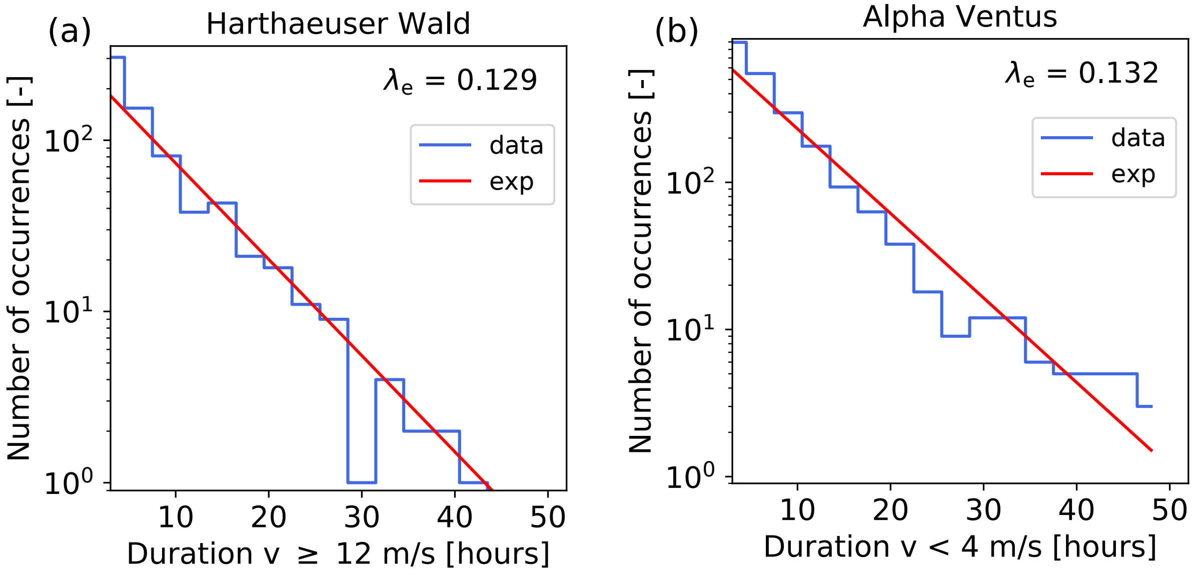

Supplementary Figure 2. **Atypical wind conditions follow approximately exponential distributions.** a: Harthaeuser Wald is analysed for high-wind velocities $v \geq 12$m/s, while b: Alpha Ventus is used for a low-wind velocity analysis $v < 4$m/s, both based on the downscaled ERA-Interim data from 1980-2010 [1]. The blue curves give the data and the red curve depicts the most-likely exponential fits. We note that although 30 years are considered, the number of events is of the order of $N \sim 100$, so that we do not pursue a detailed analysis, e.g. splitting the data for superstatistical approaches.

The main text demonstrated that low-wind persistence statistics is better described by q-exponentials than exponential functions for low-wind locations such as Harthaeuser Wald. Similarly, we find that persistence statistics of high-wind is also better described by q-exponentials for high-wind locations such as Alpha Ventus. We illustrate this by investigating high-wind persistence statistics of the 9 sample locations from Supplementary Fig. 1 in Supplementary Fig. 3.

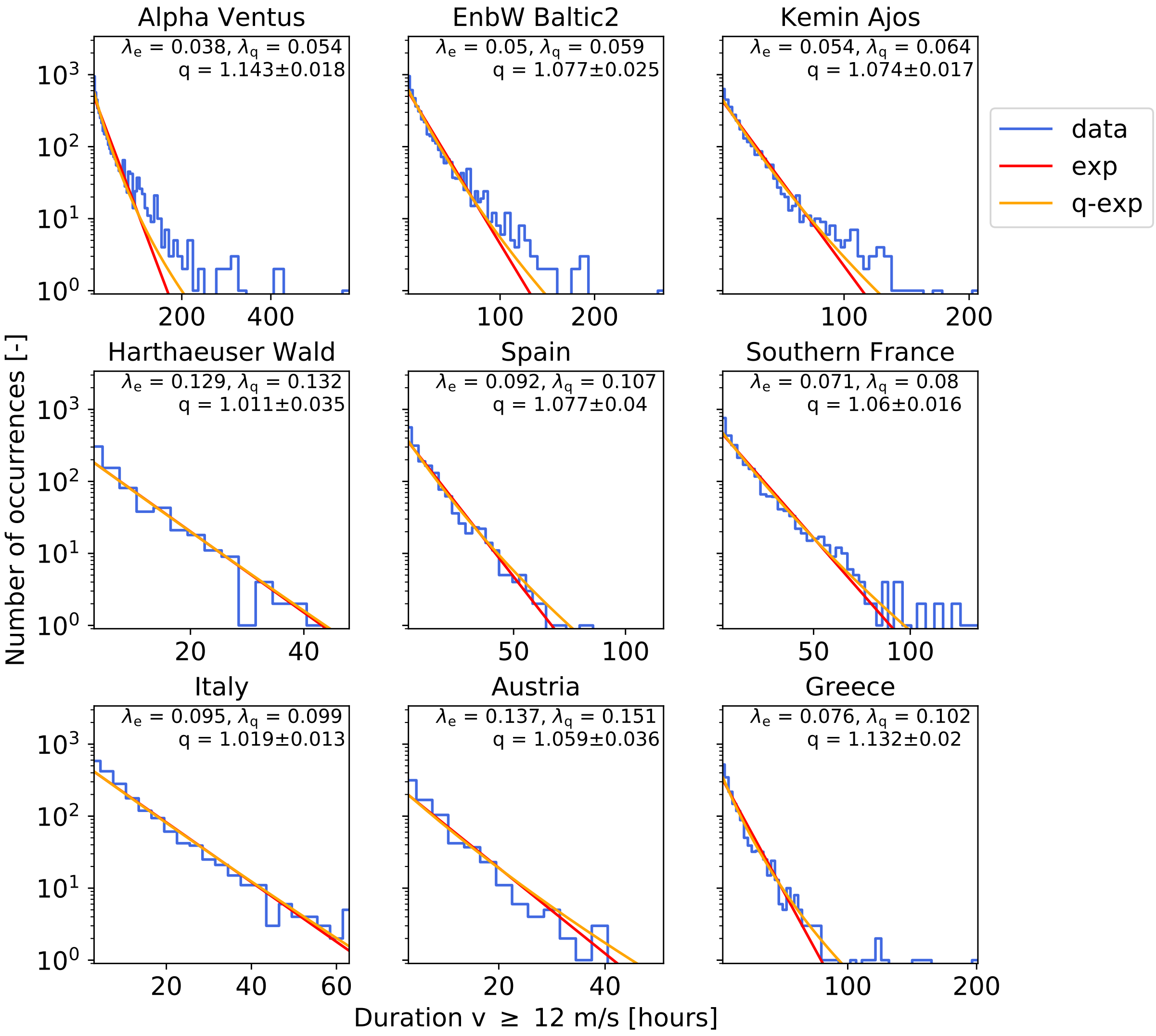

Supplementary Figure 3. **Distributions are not strictly exponential but better described by q-exponentials for high-wind.** Wind persistence statistics (blue) is shown with the best-fitting exponential (red) and q-exponential distributions (orange) for 9 selected locations, based on the downscaled ERA-Interim data [1]. The q-values are determined by using the kurtosis of the data, see eq. (1). Note that the maximum q-value derived this way is $q_{max} = 1.2$.

SUPPLEMENTARY NOTE 3

q-Parameters for Europe: Off and on-shore

In the main text, we analyzed the wind power generation per country and observed heavy tails in the wind power persistence statistics, similar to the wind velocity persistence statistics. Is it possible to split the power generation into more homogeneous chunks to explain the heavy tails as a superposition of exponentials [2, 3]? For example, we could make use of the separation within the data set into onshore and offshore wind generation.

We compare the q-values for the aggregated data (on- and offshore combined) with the onshore and the offshore data individually for France, Great Britain and Germany, see Supplementary Table II. We do not notice a clear trend that determines the heavy tails of the distributions, i.e., the full data as well as the subsets are heavy-tailed.

Supplementary Table II. q-values for different regions, considering on- and offshore generation separately as well as aggregated. The q-values are computed based on the kurtosis of the power generation statistics [4].

Region/q	$q_{\text{Aggregated}}$	q_{Offshore}	q_{Onshore}
Germany	1.05823	1.05714	1.07016
France	1.07918	1.06426	1.07923
GB	1.09204	1.10208	1.09646

SUPPLEMENTARY NOTE 4

Further superstatistical analysis

When following the super-exponential approach in the main text, we split the data based on small bins of homogeneous f-parameters for Harthaeuser Wald. Here, we show the same analysis applied to the high-wind location Alpha Ventus using high-wind statistics, i.e., $v \geq 12$m/s, see Supplementary Fig. 4. We again observe that individual bins are better described by exponentials [5] than the full data (lower q-values).

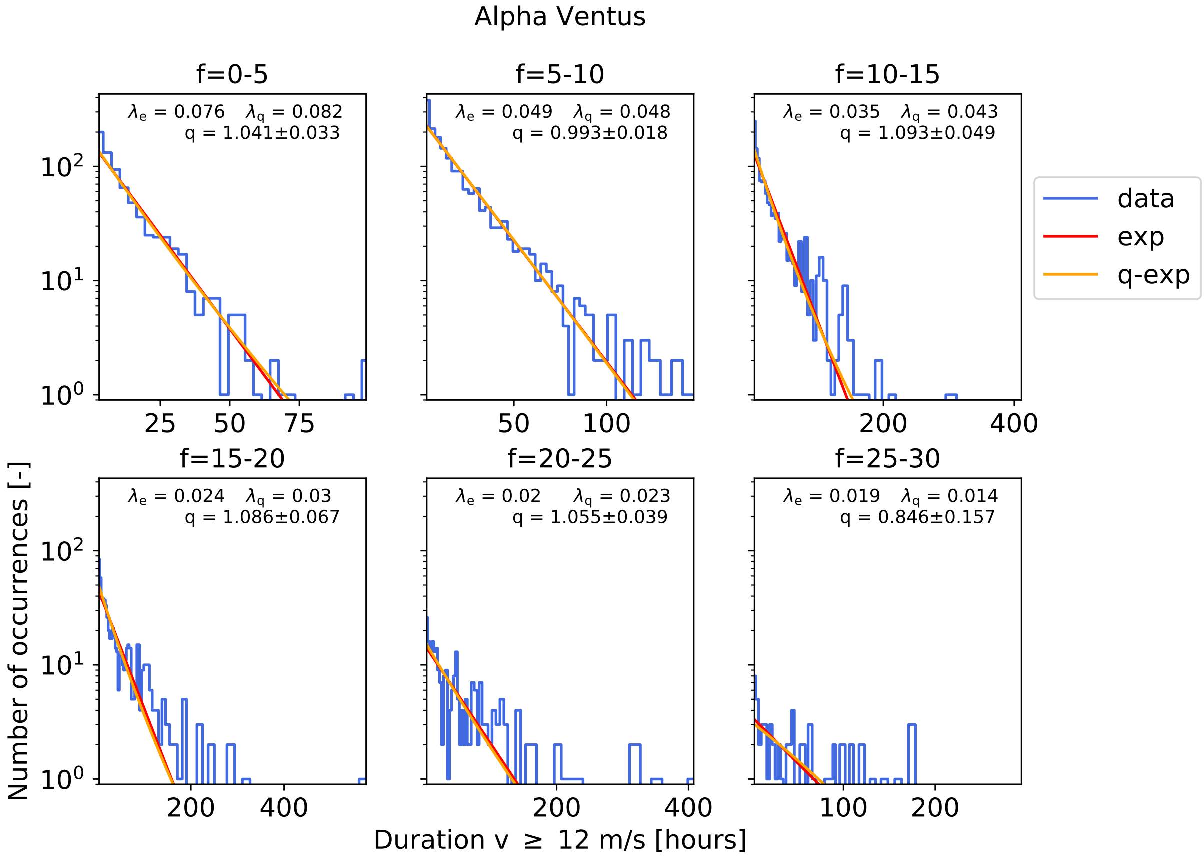

Supplementary Figure 4. **Persistence statistics is approximated by exponentials if the data set is divided by homogeneous f-parameters.** High-wind velocity statistics, $v \geq 12$m/s, is analyzed at Alpha Ventus, based on the downscaled ERA-Interim data [1], conditioning the statistics on small bins of homogeneous f-parameter (in units of hPa per $1000km$). Performing both an exponential and a q-exponential fit, we notice that each fit is close to an exponential. The q-value is determined by using the kurtosis of the data, see eq. (1). Note that the maximum possible q-value derived this way is $q_{max} = 1.2$.

As often mentioned, the kurtosis as a function of q diverges at $q = 1.2$. Many heavy-tailed distributions, e.g. Lévy-stable or q-exponential distributions no longer have their higher moments defined for certain parameters [6]. If we compute the n-th centralized moment as

$$\mu_n := \int_{-\infty}^{\infty} (x-\mu)^n \, p(x) \, \mathrm{d}x, \tag{2}$$

with mean μ, then the kurtosis is given as

$$\kappa = \frac{\mu_4}{\mu_2^2}. \tag{3}$$

If the distribution has heavy tails, then the probability density function $p(x)$ does not decay fast enough, so that the integrand $(x-\mu)^n \, p(x)$ is too large for large values of $|x|$. Therefore, the integral for μ_4 no longer exists. For even heavier tails, such as in Lévy-stable distributions, the variance or even the mean may no longer be defined [6].

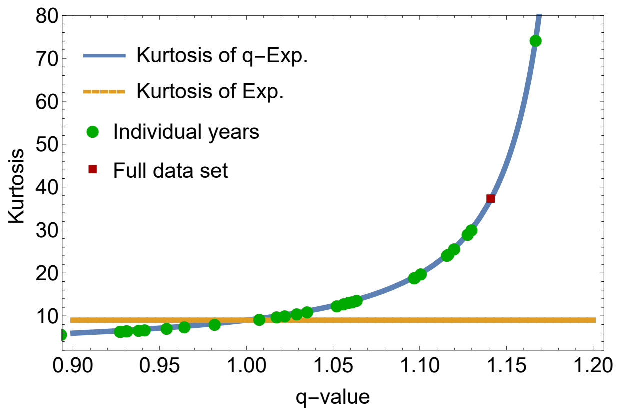

Supplementary Figure 5. **Wind velocity statistics robustly returns q-values above 1.** High-wind velocity persistence statistics, $v \geq 12$ m/s, is analyzed at Alpha Ventus, based on the downscaled ERA-Interim data from 1980-2010 [1]. The kurtosis is plotted as a function of the q-value, eq. (1), compared to the constant exponential kurtosis of $\kappa = 9$. The points give the individual kurtosis (green) values, when splitting the data into 31 equally sized bits, based on the 31 recorded years. For reference, the kurtosis of an exponential distribution, namely $\kappa = 9$ is included as the orange line. Even when splitting the data into smaller chunks, most values return a q-value larger than 1.

We visualize the divergence of the kurtosis as a function of q together with kurtosis values of a yearly disaggregation of the Alpha Ventus high-wind data in Supplementary Fig. 5.

Furthermore, we repeat the box plot from the main text, this time using the downscaled ERA-Interim data at Harthaeuser Wald, comparing yearly division with conditional division and an artificial Poisson process. Similar to the main text, we note that the q-values of yearly subsets are larger than when conditionally splitting the data (Supplementary Fig. 6). In addition, we notice that a few large q-values in the whiskers of the box-plot seem to determine the overall q-value of the full data set.

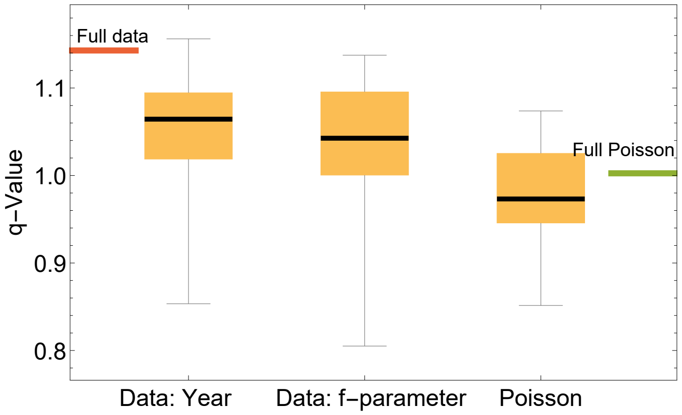

Supplementary Figure 6. **Conditioning data to f-parameters approximates Poissonian statistics.** Low-wind velocity statistics, $v < 4$m/s, is analyzed at Harthaeuser Wald, based on the downscaled ERA-Interim data from 1980-2010 [1]. The data set is either split for each year, or conditionally so that 31 similarly sized subsets are created, each with approximately homogeneous f-parameter. Finally, this is compared to an artificial Poisson distribution, see Methods of the main text for details. The colored lines give the q-value of the full data set and the full artificial Poisson data set. The box plot gives the median as a black line, the 25% to 75% quartile as yellow box and minimum and maximum value as whiskers.

Next, we investigate superstatistical differences between Harthaeuser Wald and Alpha Ventus, using their typical wind conditions respectively. In the main text we introduced the distribution $p(d)$ of the persistence statistics/waiting time d as

$$p(d) = \int_0^\infty g(\lambda_e) p(d|\lambda_e) \mathrm{d}\lambda_e, \quad (4)$$

where $p(d|\lambda_e)$ follows an exponential distribution for fixed λ_e. Following superstatistical theory, $g(\lambda_e)$ should follow

a χ^2 or Log-Normal distribution [2, 3] to analytically recover q-exponentials for $p(d)$. Indeed, we observe that λ_e is well-approximated by a Log-Normal distribution (Supplementary Fig. 7).

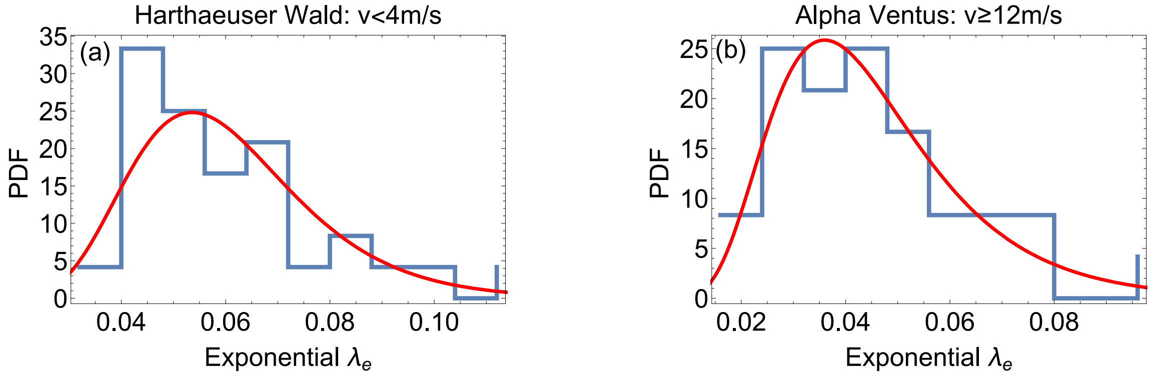

Supplementary Figure 7. **The distribution of λ_e approximately follows a log-normal distribution.** The downscaled ERA-Interim data set [1] is split so that 31 similarly sized subsets are created, each with approximately homogeneous f-parameter. a: Harthaeuser Wald for $v < 4$m/s, b: Alpha Ventus for $v \geq 12$m/s. The blue line gives the histogram and the red line gives the most-likely log-normal fit. Panel (b) is also shown in the super exponential analysis in Fig. 5e in the main text.

Furthermore, we investigate the dependency of the exponential decay rate λ_e on the f-parameter in Supplementary Fig. 8. For the low-wind location Harthaeuser Wald, λ_e tends to increase with increasing f-parameter while it decreases for the high-wind location Alpha Ventus. Interestingly, the f-decomposition does not work as well for the low-wind persistence statistics: In Supplementary Fig. 8a, the dependency f-parameter-λ_e is not monotonic but first decreases and then increases. In addition, the super-positioned exponentials do not fit the q-exponential as well as in the case of the high-wind statistics (see below).

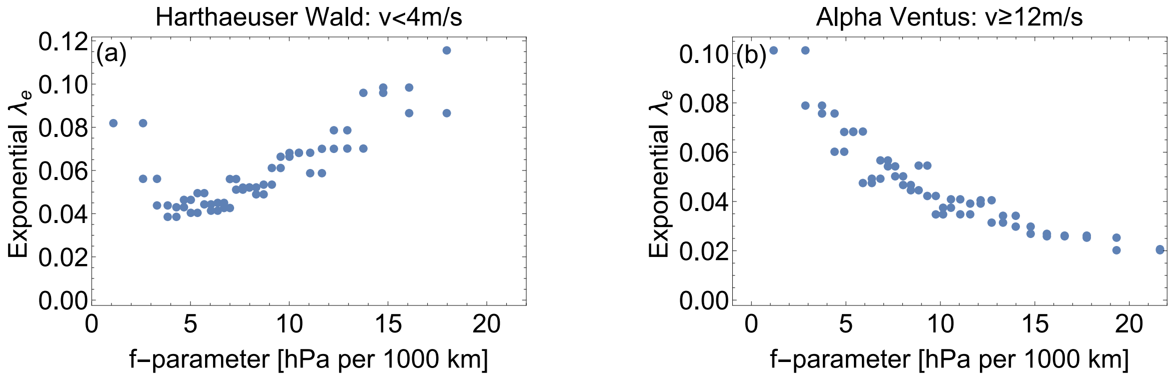

Supplementary Figure 8. **The exponential decay rate λ_e depends on the f-parameter.** The downscaled ERA-Interim data set [1] is split so that 31 similarly sized subsets are created, each with approximately homogeneous f-parameter, see main text Methods for details. a: Harthaeuser Wald for $v < 4$m/s, b: Alpha Ventus for $v \geq 12$m/s. We note that λ_e decreases with the f-parameter in the case of a high-wind location (Alpha Ventus) and increases for a low-wind location (Harthaeuser wald).

Finally, we demonstrate that q-exponentials can be approximated by superimposing exponentials, as discussed in the main text, see Supplementary Fig. 9. When superimposing different exponential distributions to generate Supplementary Fig. 9, we used the following procedure. We split the full data set into M subsets of approximately constant f-parameter. Let $S_{\text{exp},\, i}$ be the characteristic function of the ith subset for a fixed f-parameter. And let N_i be the number of data points within the set. Then, we compute the characteristic function of the superimposed exponential as

$$S_{\text{super-exp}} = \frac{\sum_{i=1}^{M} N_i S_{\text{exp},\, i}}{\sum_{i=1}^{M} N_i}. \tag{5}$$

The probability density function is then obtained as the Fourier transform of the characteristic function.

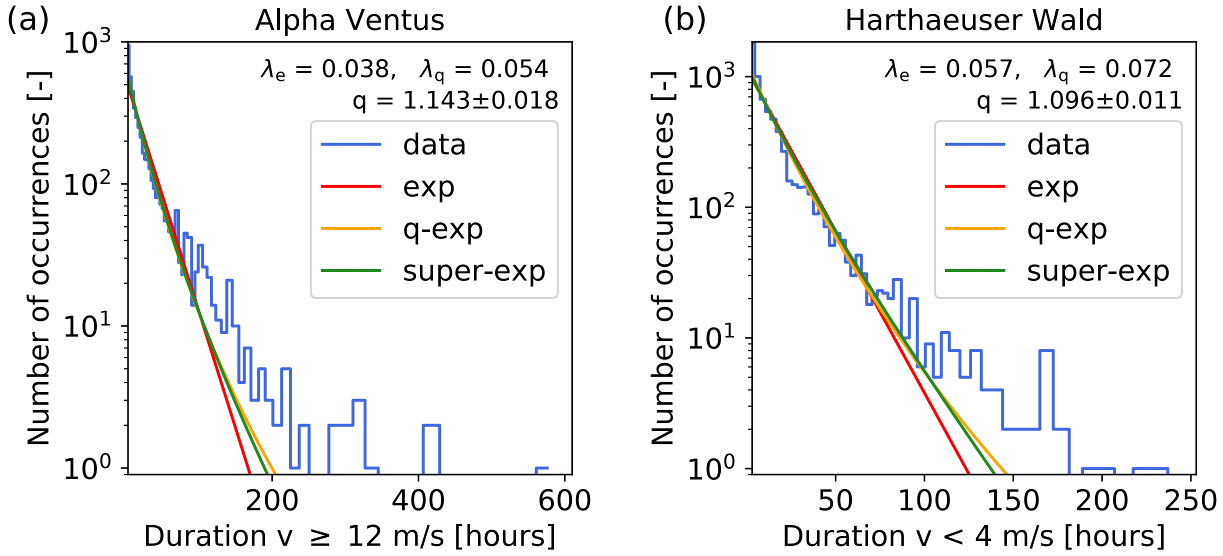

Supplementary Figure 9. **Superimposing exponential distributions approximates q-exponentials using f-parameter.** Harthaeuser Wald (a) is analysed for low-wind velocities $v < 4$ m/s, while Alpha Ventus (b) is used for high-wind velocity analysis $v \geq 12$ m/s, both based on the downscaled ERA-Interim data from 1980-2010 [1]. For each data set, a superposition of exponentials is formed, denoted as *super-exp*, based on the individual exponential distribution for different f-parameters. The superimposed exponentials approximate the directly fitted q-exponentials in particular for the high-wind persistence.

So far, we considered different f-parameters as a criterion to split the data. Instead, we could choose bins based on constant CWT direction, see Supplementary Fig. 10 for the results. Superimposing these exponentials gives an approximation to the q-exponential but is still much closer to the original exponential fit, especially for Alpha Ventus, which is heavily dominated by west CWT for high-wind speeds. The CWT directions considered here are 'North', 'North-East', 'East', 'South-East', 'South', 'South-West', 'West', 'North-West', 'West', 'North-West', 'Cyclonic' and 'Anti-cyclonic'.

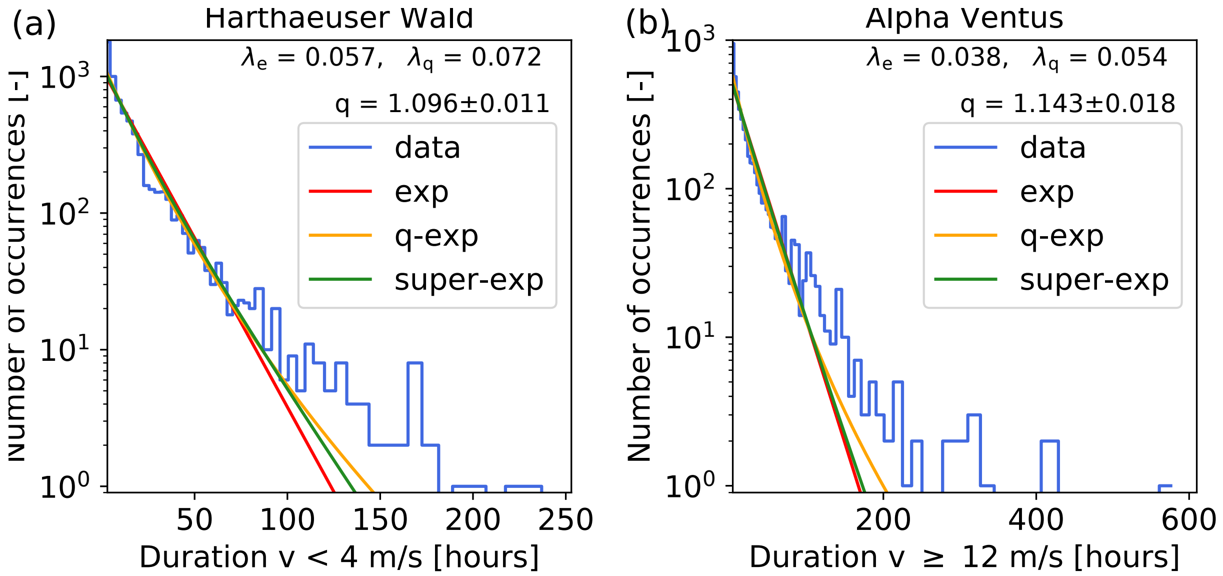

Supplementary Figure 10. **Superimposing exponential distributions approximates q-exponentials using CWT directions.** Harthaeuser Wald (a) is analysed for low-wind velocities $v < 4$ m/s, while Alpha Ventus (b) is used for high-wind velocity analysis $v \geq 12$ m/s, both based on the downscaled ERA-Interim data from 1980-2010 [1]. For each data set, a superposition of exponentials is formed, denoted as *super-exp*, based on the individual exponential distribution for different CWT directions. The superimposed exponentials approximate the directly fitted q-exponentials not as well as the superstatistical approach based on the f-parameter.

SUPPLEMENTARY NOTE 5

Further synoptic analysis

To better understand long persistence periods, we analyzed the average and standard deviation of the mean sea level pressure (MSLP) in the main text. Here, we present and discuss individual snapshots of the high-wind situation 13 November to 08 December 2006 (609 hours: HP1) and of the high-wind situation 10 October to 28 October 1983 (435 hours; HP2). The synoptic situation for early 1990 (HP3) is similar to HP1 and has been described in [7]. Therefore, only HP1 and HP2 are discussed in detailed. The HP1 period (Supplementary Fig. 11) is characterised by the recurrent presence of a trough over the North Atlantic and dominant and strong south-westerly flow over Western Europe. While the large-scale flow changes little during this period (typically changing from westerly to south-westerly flow and back), pressure gradients and thus the strong winds remain. Embedded in this strong flow, distinct low pressure systems (secondary lows) pass over the British Isles and the North Sea. The presence of high pressure systems over the subtropic North Atlantic and Southern Europe was important to maintain the strong pressure gradients throughout this period. As a result, the main MSLP fields changed only little during the HP1 period.

The synoptic conditions for HP2 are more diverse (Supplementary Fig. 12). First, there is a stronger interplay between high and low pressure centers over Western Europe, which leads to the strong variance identified in Figure 8e of the main text. Second, the role of the high pressure ridges (extending from the Iberian Peninsula towards Central Europe) and highs (either over the UK or Central Europe) is very dominant, leading to a recurrent anticyclonic flow. Given the juxtaposition of this high pressure system with the passage of low pressure centers to the North, the recurrent anticyclonic and high pressure gradient conditions remain dominant over the North Sea for about three weeks, with short intrusions by cyclonic systems (e.g. 15.10.1983). Overall, this again highlights the diverse synoptic conditions leading to long persistence periods.

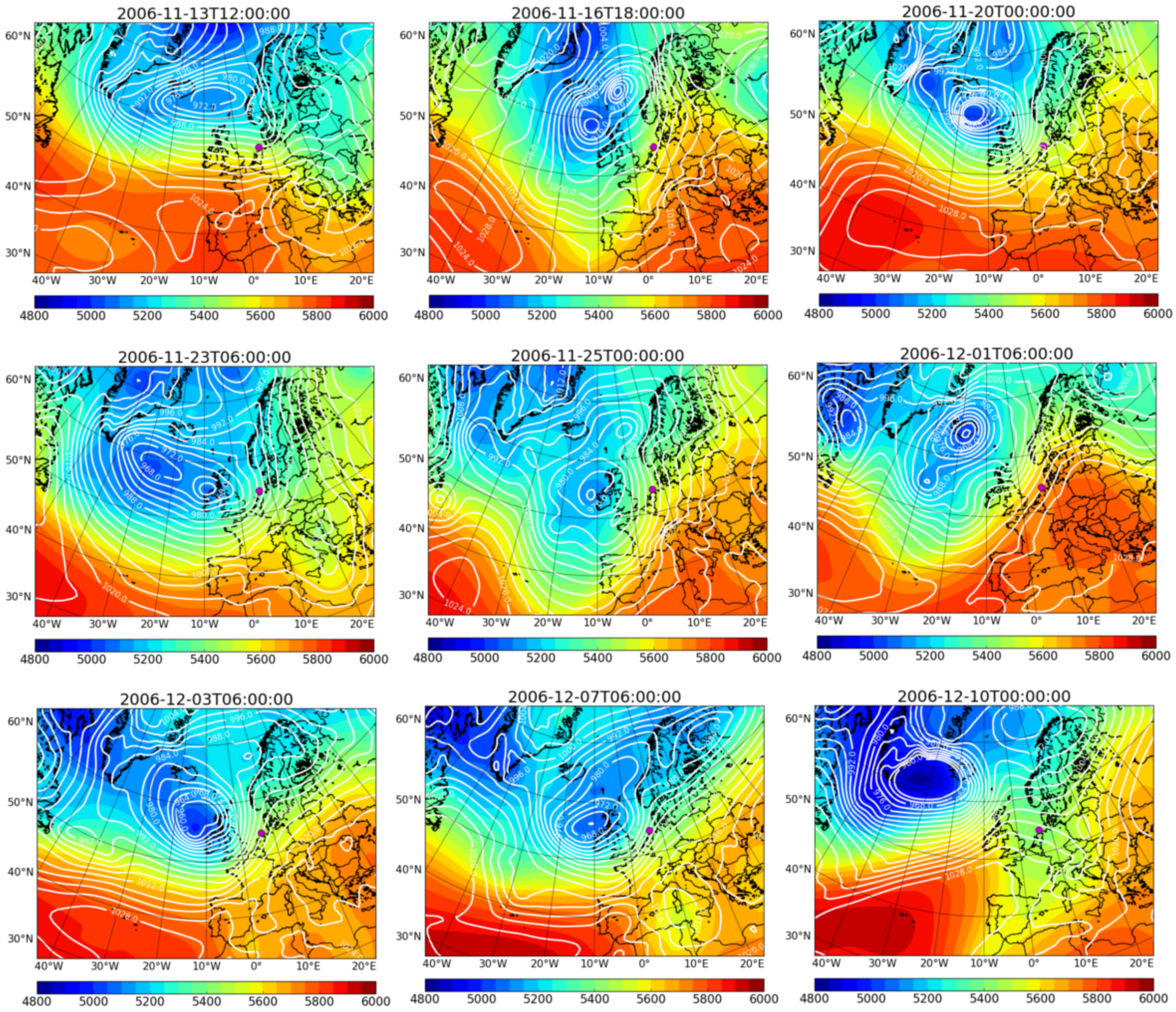

Supplementary Figure 11. **The MSLP profile does not change qualitatively during HP1 with a constant trough.** The nine panels illustrate the large scale atmospheric conditions at different time stamps of this extremely long high-wind period. The contours show the current MSLP in hPa while the shading shows the 500hPa geopotential height in meter. The magenta dot shows the location of Alpha Ventus. Maps were created using Python 2.7.12: `https://www.python.org/`.

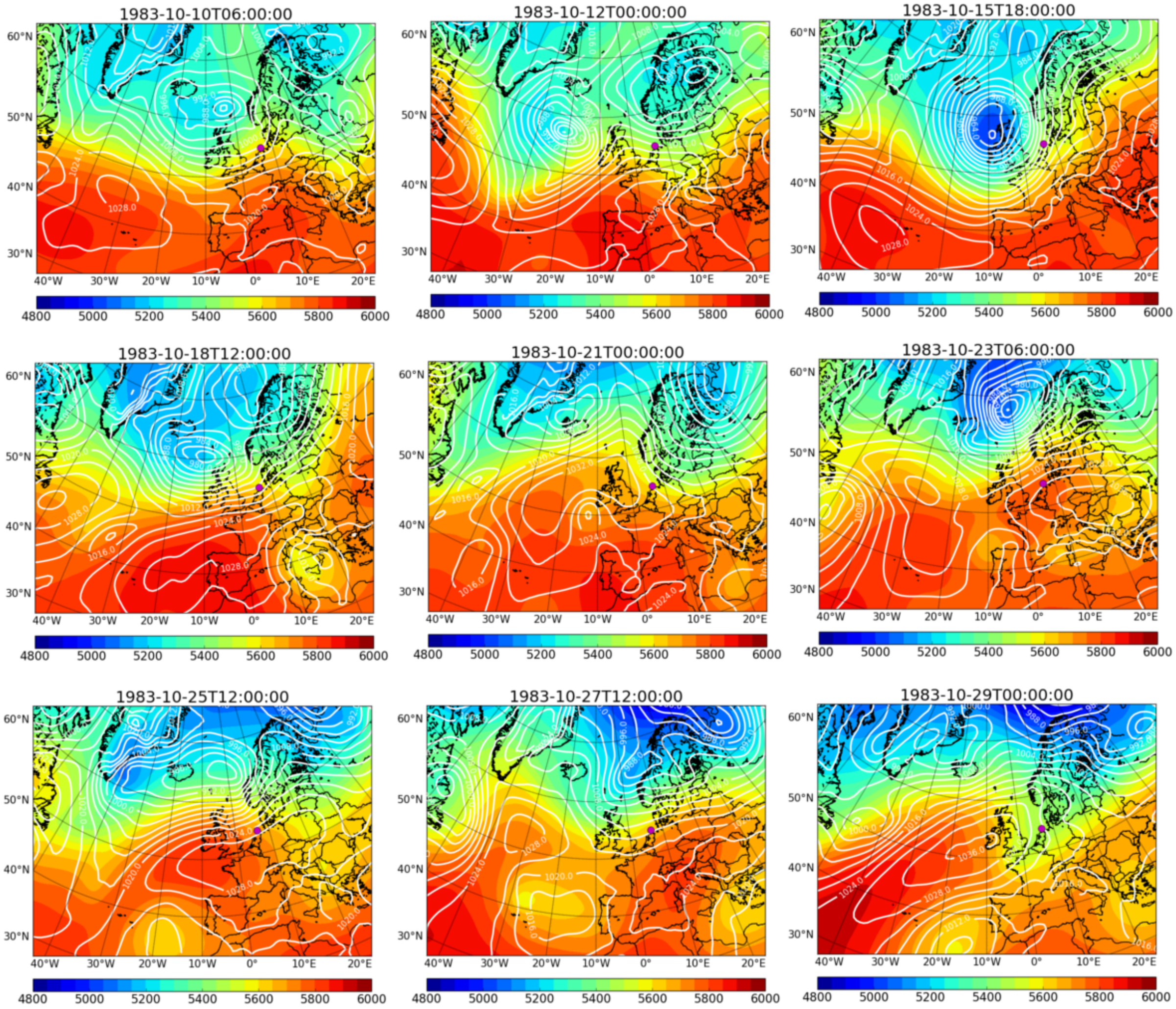

Supplementary Figure 12. **Distinct synoptic systems pass by Alpha Ventus during HP2.** The nine panels illustrate the large scale atmospheric conditions at different time stamps of this extremely long high-wind period. The contours show the current MSLP in hPa while the shading shows the 500hPa geopotential height in meter. The magenta dot shows the location of Alpha Ventus. Maps were created using Python 2.7.12: `https://www.python.org/`.

SUPPLEMENTARY NOTE 6

Time resolution, cut-off speed and likelihoods

The main text presented an analysis of wind velocities based on the ERA-Interim data set [1] using a 3h time resolution and neglected effects of wind velocities so high that the turbine has to shut down [8, 9]. Furthermore, we compared exponential and q-exponential plots visually but did not show a quantitative comparison. Here, we supplement the main text analysis with additional comparisons and plots.

First, let us consider that the time resolution of the wind data was no longer at 1 data point every 3 hours but coarser, e.g. we would only use every second data point available to us, resulting in an effective 6h time resolution. Would our analysis change? Do more drastic changes occur when using a 12 h resolution? To answer these questions, we repeat our computation of the q-parameter for different effective time resolutions for both Alpha Ventus and Harthaeuser Wald. To also investigate the effect of a finer time resolution, we apply interpolation.

A coarser time resolution tends to reduce the q-value slightly. Fewer data points in this case imply lighter tails (see Supplementary Fig. 13). This trend is clear for Alpha Ventus, while the 12h time resolution for Harthaeuser Wald reports increased tails again, which are still below the original 3h resolution values. In any case, the deviation of the computed q value as a function of the time resolution is comparable to the uncertainty of the estimation itself. More importantly, even within all error margins, we robustly observe heavy tails and q-values larger than 1.

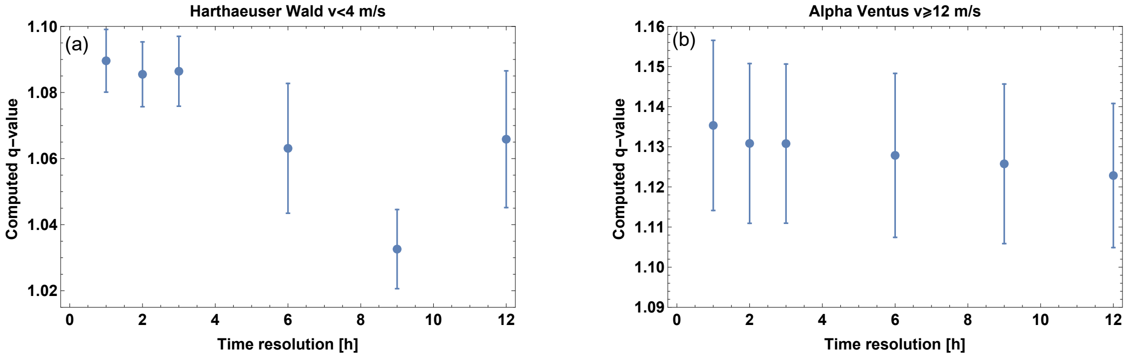

Supplementary Figure 13. **Reduced time resolution does not change our results significantly.** The downscaled ERA-Interim data set [1] is used to determine the q-value as a function of the effective time resolution. Error bars give the standard deviation of the estimated based on bootstrapping, see main text Methods for details. a: Harthaeuser Wald for $v < 4$m/s, b: Alpha Ventus for $v \geq 12$m/s. While the tails tend to become lighter for coarser resolution, the effect is small.

Furthermore, we might consider a maximum wind velocity at which a wind turbine can generate power. Very high velocities typically lead to a shutdown of the wind turbine, thereby introducing an effective cut-off velocity. The precise cut-off will depend on the turbine [8] so that we consider a range of possible values for which instead of generating its maximum power, no power would be generated. We investigate the statistics at a particular high-wind location, Alpha Ventus, and set the velocity to zero if it surpasses the cut-off velocity $v_{\text{cut-off}}$. Again, our estimates of the q-value only change slightly when introducing a cut-off, see Supplementary Fig. 14. Naturally, a very low cut-off wind speed of $v_{\text{cut-off}} \sim 15m/s$ will reduce the heavy tails and thereby q substantially because many high wind velocities will be cut off, as we only consider wind velocities as high if $v > v_{\text{High}} = 12m/s$. However, it is more realistic to assume a cut-off wind speed of $v_{\text{cut-off}} \sim 25m/s$ [8]. For this cut-off speed, the statistics of the original distributions without cut-off and with cut-off are essentially the same.

Finally, we noticed how q-exponentials visually are a better fit to the wind duration data than exponentials are based on several plots in the main text and this Supplementary Information. We quantify this statement by computing the likelihood ratios of the exponential and the q-exponential distribution for Alpha Ventus and Harthaeuser Wald: Given a probability density function $p(x)$ and a data set $Y = \{y_1, y_2, ..., y_N\}$, we calculate the likelihood that Y is drawn from the distribution p by calculating

$$L_{p,Y} = \Pi_{i=1}^{N} p(y_i). \tag{6}$$

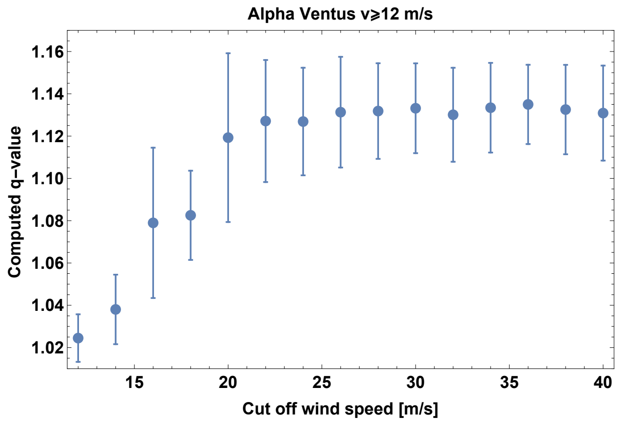

Supplementary Figure 14. **Introducing a cut-off wind speed has a minor impact on our results.** The downscaled ERA-Interim data set [1] at Alpha Ventus for $v \geq 12$ is used to determine the q-value as a function of a cut-off wind speed for which we set the velocity to zero instead. Error bars give the standard deviation of the estimated based on bootstrapping, see main text Methods for details.

The maximum likelihood estimate is based on comparing at least two different distributions, e.g., $p_1(x)$ and $p_2(x)$ by computing the likelihoods for both distributions. Next, we have a look at the logarithm of the likelihood ratio

$$\log_{10}\left(\frac{L_{p_1,Y}}{L_{p_2,Y}}\right), \tag{7}$$

which is the most powerful tool to distinguish two distributions [10].

Comparing exponentials and q-exponentials at Alpha Ventus and Harthaeuser Wald, the respective results are

$$\log_{10}\left(\frac{L_{p_{q-exp},Y}}{L_{p_{exp},Y}}\right)_{\text{Alpha Ventus}} \approx 125, \tag{8}$$

$$\log_{10}\left(\frac{L_{p_{q-exp},Y}}{L_{p_{exp},Y}}\right)_{\text{Harthaeuser Wald}} \approx 110. \tag{9}$$

So the q-exponentials are a much more likely fit to the original data than exponentials are.

SUPPLEMENTARY NOTE 7

Long-range correlations in the time series

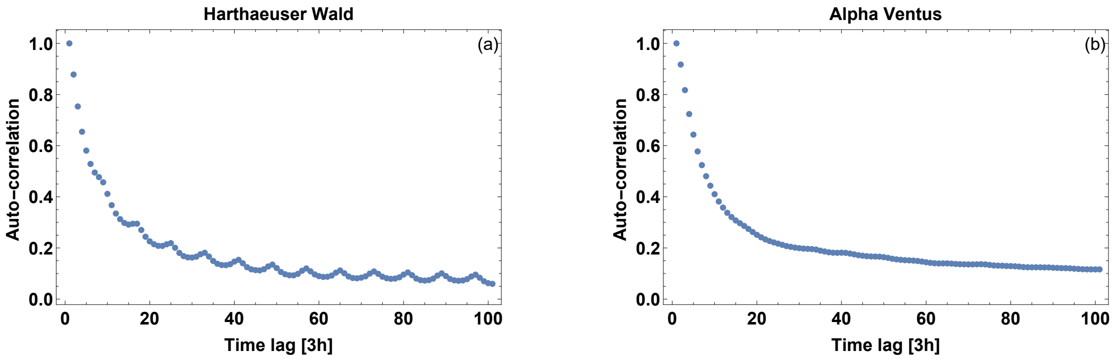

Supplementary Figure 15. **Autocorrelation stabilizes to non-zero values.** The downscaled ERA-Interim data set [1] is used to determine the autocorrelation as a function of the time lag. a: Harthaeuser Wald, b: Alpha Ventus. Following the exponential decay of the autocorrelation, we notice that it stabilizes to a non-zero value which is kept for long time lags.

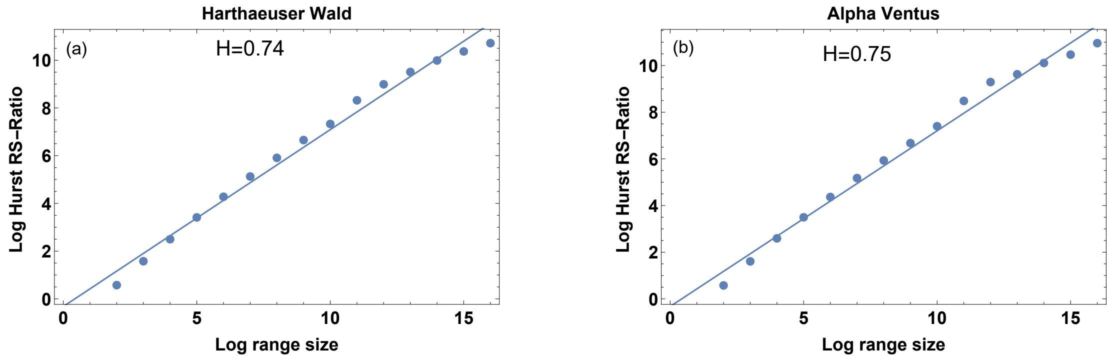

Supplementary Figure 16. **Hurst exponent analysis reveals long-range correlations of the time series.** The downscaled ERA-Interim data set [1] is used to determine the Hurst exponent [11]. Both axes use a log-scale and the estimated expoinent is given in each plot. a: Harthaeuser Wald, b: Alpha Ventus.

We might take an alternative perspective when investigating the time series: Long persistent periods of high or low wind indicate that given a high wind speed, we expect the wind speed to stay very high for the next hours. This should be reflected in a positive long-range correlation of the time series [12]. Indeed, plotting the autocorrelation functions for both Alpha Ventus and Harthaeuser Wald, we notice an initial decay of the autocorrelation, which then stabilizes to a non-zero value (Supplementary Fig. 15). This long-term correlation is likely caused by seasonal effects, i.e., wind speeds are higher in winter than in summer.

To further investigate long-range correlations, we compute the Hurst exponent [11]. Again, we observe a significant positive long-range correlation (Supplementary Fig. 16). The Hurst exponents are determined as $H \approx 0.75$ for both Alpha Ventus and Harthaeuser Wald. Compared to an uncorrelated value of $H_{\text{uncorrelated}} = 0.5$, this implies that high wind velocities are much more likely followed by high velocities than by low ones and vice versa. Overall, this correlation fits perfectly to the observed heavy tails of the persistence statistics.

SUPPLEMENTARY NOTE 8

Impacts of persistence statistics on storage dimensioning

We have highlighted the heavy tails in the wind persistence statistics. These tails will have to be considered when dimensioning storage facilities of future energy systems. The storage has to be large enough to compensate for fluctuations and should also be sufficient for all but the most extreme events. Here we demonstrate how more pronounced heavy tails, measured via the q-parameter, lead to larger necessary storage facilities. The additional storage demand grows rapidly with q.

We model the storage requirements as follow. The storage has to balance time periods with predominant low-wind states, without considering long-range transmission, photovoltaic, etc. We generate persistence statistics for $q = 1$ using a single Poissonian process, based on values recorded at Harthaeuser Wald. For $q > 1$, we combine 30 Poissonian processes with different rates into one aggregated process. Thereby, the persistence statistics of the aggregated process do no longer follow an exponential but a q-exponential distribution [2, 3]. To determine the different decay rates, we use a log-normal distribution

$$p(x) = \frac{1}{\sqrt{2\pi}x\sigma} \exp\left(-\frac{\left(-\mu + \log(x)\right)^2}{2\sigma^2}\right) \tag{10}$$

with fixed $\mu = -3$ and several $\sigma \in \{0.1, 0.2, ..., 1\}$ to cover multiple q-values. See also Supplementary Note 4 for log-normal fits for the different exponential rates observed at Alpha Ventus and Harthaeuser Wald. With the synthetic persistence statistics, we now have to define the storage needs.

We expect short periods of low wind to be balanced by daily options and only long durations with low wind to require back-up storage, see Supplementary Fig. 17. In particular, we assume that any shortage of wind lasting for less than 24 hours is compensated by short-term options. For any longer low-wind states, we quantify the storage requirements in terms of 1 day (24 hour) storage and normalize the storage requirements with respect to the $q = 1$ case. Furthermore, we simplify the analysis by assuming the storage capacity is fully charged directly after a low-wind state. We plot the required storage needs to cover 90% or 99% of time with low-wind states in Supplementary Fig. 18 (a) and (b) respectively.

Heavy tails of the persistence statistics, i.e. $q > 1$, do indeed lead to higher storage requirements. If the system only needs to operate about 90% of the time during low-wind states, the storage requirement doubles for very heavy tails and a q-parameter of $q_{90\%\ \mathrm{Double}} \approx 1.16$. If we operate a more critical system and demand secure operation for at least 99% of low-wind states, the storage needs grow faster with heavier tails. Doubling the capacity is necessary for much more moderate q-parameters of $q_{99\%\ \mathrm{Double}} \approx 1.12$ and for even larger q-parameters the necessary storage capacity continues to grow rapidly. Hence, for non-critical systems, the standard, non-heavy-tail exponential estimates might be sufficient, while critical systems require a considerably higher storage capacity.

Critically, the risk assessments should not be quantified via multiples of the standard deviations, such as σ, 2σ, etc. These estimates work for standard Gaussian distributions. Here, we observe heavy tails and instead use measures such as securing 90% or 99% of the low-wind states.

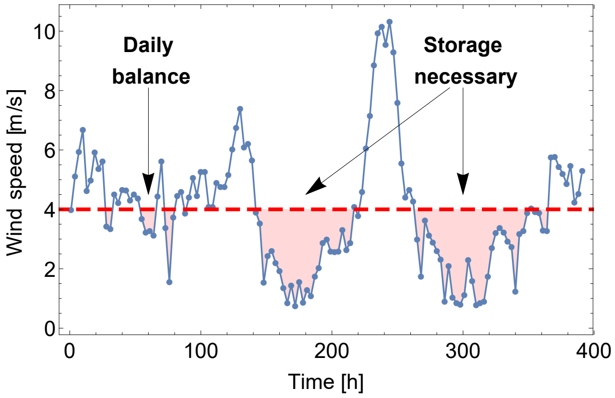

Supplementary Figure 17. **Short periods of low wind are likely compensated by short-term measures, while storage becomes necessary to balance long periods with low wind.** We plot wind velocities at Harthaeuser Wald, based on the downscaled ERA-Interim data from 1980-2010 [1] to illustrate our assumptions for estimating storage requirements. Periods of less than 24 hours are assumed to be balanced by daily options and additional storage needs are quantified in mutiples of days, i.e., 24 hours.

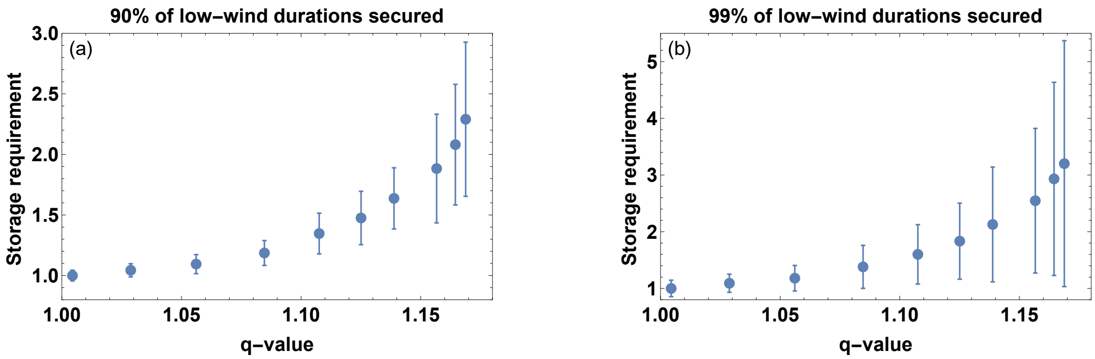

Supplementary Figure 18. **With increasing heavy tails, storage needs grow.** The necessary storage capacity is plotted as a function of the q-parameter of the underlying persistence statistics. For a given q-parameter we use 100 different realizations and report both the mean and the standard deviation in the plot. To realize a 100% reliable system, the storage would have to cover the single longest low-wind state, which is typically unknown. Instead, we give storage requirements to cover 90% (a) or 99% (b) of the low-wind durations. Note the difference in the vertical scale between (a) and (b). All values are normalized to the $q = 1$ case.

SUPPLEMENTARY REFERENCES

[1] Dee, D. P. *et al.* The ERA-Interim reanalysis: Configuration and performance of the data assimilation system. *Quarterly Journal of the Royal Meteorological Society* **137**, 553–597 (2011).
[2] Beck, C. Dynamical foundations of nonextensive statistical mechanics. *Physical Review Letters* **87**, 180601 (2001).
[3] Beck, C. & Cohen, E. G. D. Superstatistics. *Physica A* **322**, 267–275 (2003).
[4] Staffell, I. & Pfenninger, S. Using bias-corrected reanalysis to simulate current and future wind power output. *Energy* **114**, 1224–1239 (2016).
[5] Ross, S. M. *Introduction to Probability Models* (Academic press, 2014).
[6] Samorodnitsky, G. & Taqqu, M. S. *Stable Non-Gaussian Random Processes. Stochastic Models with Infinite Variance* (Chapman and Hall, 1994).
[7] Pinto, J. G. *et al.* Large-scale dynamics associated with clustering of extratropical cyclones affecting Western Europe. *Journal of Gephysical Research - Atmospheres* **119**, 13,704–13,719 (2014).
[8] Ackermann, T. *Wind Power in Power Systems* (John Wiley & Sons, 2005).
[9] Manwell, J. F., McGowan, J. G. & Rogers, A. L. *Wind Energy Explained: Theory, Design and Application* (John Wiley & Sons, 2010).
[10] Bohm, G. & Zech, G. *Introduction to statistics and data analysis for physicists* (DESY, 2010).
[11] Hurst, H. E. Long-term storage capacity of reservoirs. *Trans. Amer. Soc. Civil Eng.* **116**, 770–799 (1951).
[12] Anvari, M. *et al.* Stochastic nature of series of waiting times. *Physical Review E* **87**, 062139 (2013).
